# Management of congenital aortic and truncal valve disease: Art or science?

**DOI:** 10.1016/j.xjon.2026.101678

**Published:** 2026-02-13

**Authors:** Amine Mazine, Edward Buratto, Igor E. Konstantinov

**Affiliations:** a Department of Cardiothoracic Surgery, Royal Children's Hospital, Melbourne, Australia; b Department of Paediatrics, University of Melbourne, Melbourne, Australia; c Heart Research Group, Murdoch Children's Research Institute, Melbourne, Australia; d Melbourne Centre for Cardiovascular Genomics and Regenerative Medicine, Melbourne, Australia

**Keywords:** congenital aortic, truncal valve disease, reconstruction

**Figure:**
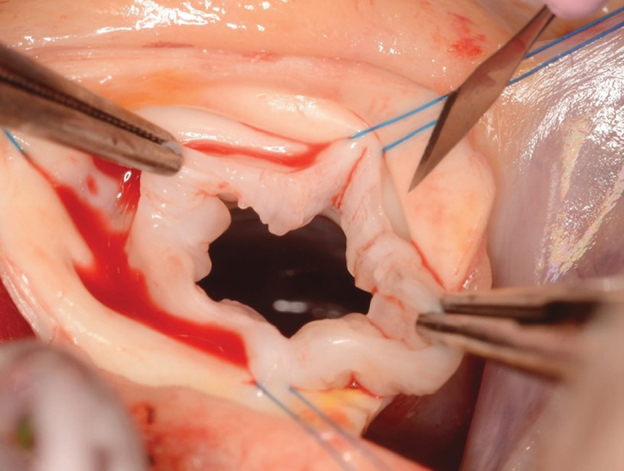
Bicuspidization of a unicuspid aortic valve in a 2-year-old child.

**Central Message**

Anatomy-guided reconstruction that preserves native valves can delay replacement and improve outcomes in congenital aortic and truncal valve disease despite limited trial evidence.

Congenital heart surgery remains one of the few areas of medicine in which Level A evidence, defined as high-quality data from multiple randomized controlled trials or their meta-analyses, is rarely obtainable.^1^ Small patient populations, marked anatomical heterogeneity, and ethical and logistical barriers limit the feasibility of large prospective trials. As a result, clinical decision-making relies largely on retrospective series, multicenter registries, and expert consensus.

A second challenge relates to the time horizon required to assess durability. Follow-up of 20 years or longer is often needed to evaluate long-term surgical outcomes. By the time such data become available, the operative strategies used in the original cohort may have evolved or been abandoned. Thus, the most mature evidence may not accurately reflect contemporary techniques or technology.

In this environment, the “current best evidence” increasingly consists of propensity-matched analyses and systematic reviews. These methods allow historical practice patterns to be questioned and provide guidance in areas where equipoise persists.

This Invited Expert Opinion focuses on 3 such areas in the management of congenital aortic and truncal valve disease:

1. **Surgical repair versus balloon dilation** **for congenital aortic stenosis.** Although balloon dilation is the preferred initial intervention for neonates and infants at most centers worldwide, our program in Melbourne adopts a primary surgical strategy.
2. **Native aortic valve repair versus the Ross procedure.** We examine the role of valve repair as a temporizing strategy to permit somatic growth before Ross conversion.
3. **Preservation of the native truncal valve.** We emphasize standardized repair techniques to avoid neonatal valve replacement, which carries high mortality.

Across these domains, we propose that practice is shifting away from palliative or nonspecific interventions toward durable, anatomy-guided surgical solutions that preserve native valve tissue and support growth.^2^

## Surgical Repair Versus Balloon Dilation for Congenital Aortic Stenosis

The management of congenital aortic stenosis in neonates and infants remains an area of clinical equipoise. Balloon aortic dilation (BAD) is the initial therapy at most centers because it avoids sternotomy and cardiopulmonary bypass. In contrast, our institutional strategy in Melbourne favors primary surgical aortic valvuloplasty (SAV). This section reviews the evidence supporting each approach and examines how contemporary surgical methods have altered historical comparisons.

**Figure 1:**
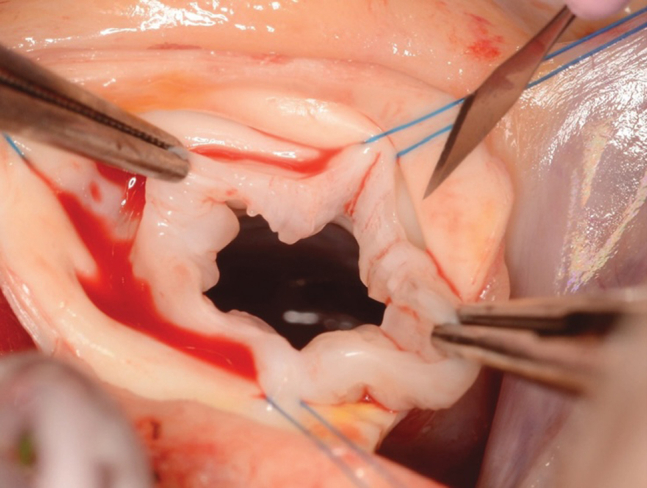
Bicuspidization of a unicuspid aortic valve in a 2-year-old child.

For many years, support for a BAD-first strategy was based largely on a 2001 Congenital Heart Surgeons’ Society multicenter study of 110 neonates in which the authors reported similar survival and freedom from reintervention after BAD and SAV.^3^ However, most surgical procedures in that era consisted of simple commissurotomy. Contemporary surgical practice incorporates leaflet thinning, resection of nodular dysplasia, and commissural resuspension.^4^ As a result, historical demonstrations of equivalence may not reflect outcomes achievable with current anatomy-guided reconstruction (Figure 1).

Our institutional preference for surgery is supported by longitudinal outcomes. In a series of 123 neonates and infants treated between 1984 and 2010, freedom from reintervention at 10 years was 65% after SAV and 27% after BAD, and BAD was an independent predictor of reintervention.^5^ Similar findings were reported in a 2016 meta-analysis of 2368 patients.^6^ Survival and freedom from aortic valve replacement (AVR) were comparable, but reintervention occurred more frequently after BAD, with 10-year freedom from reintervention of 46% after BAD versus 73% after SAV. More recent surgical series also suggest that SAV produces greater gradient reduction with a lower incidence of significant postoperative aortic insufficiency.^7^

At the same time, outcomes after BAD have improved. The use of smaller catheters, higher-pressure and lower-profile balloons, and enhanced imaging modalities has reduced procedural morbidity and improved technical success.^8^ A contemporary series from Toronto reported outcomes in 139 infants treated with modern BAD techniques, including 86% freedom from AVR at 10 years and no procedural mortality.^9^ Proponents of a BAD-first approach interpret these data as supporting BAD as a safe and effective initial palliative strategy in the absence of significant aortic insufficiency, with surgery reserved for patients whose gradients are resistant to dilation.

An important distinction between the 2 strategies is their effect on the timing of subsequent AVR. A recent study of 243 children showed that patients initially treated with BAD underwent AVR significantly earlier (median age, 12.0 years) than those treated surgically (median age, 18.5 years).^10^ In our cohort, the median time to first reintervention was 5 years after SAV and 11 months after BAD.^5^ Delaying AVR allows children to reach a larger and more stable somatic size, which may improve long-term performance of an autograft or prosthetic valve. For this reason, initial surgical repair may serve as a more effective bridge to adulthood.

## Aortic Valve Repair Versus the Ross Procedure in Children

The second major area of equipoise concerns whether to pursue native aortic valve repair as the initial strategy or to proceed directly to the Ross procedure. This decision reflects a balance between the superior early survival associated with repair and the long-term durability of the pulmonary autograft.

In our experience, native valve repair offers an important short-term survival advantage, particularly in neonates. In our institutional experience, a propensity-matched analysis of 415 children demonstrated significantly greater 15-year survival after repair than after the Ross procedure (98.0% vs 78.5%; *P* = .03).^11^ Early mortality after repair is very low, including in neonates (0.9%).^12^ Repair also functions as a strategic bridge. When an acceptable repair is achieved—generally defined as a residual gradient below 35 mm Hg with minimal insufficiency—the need for AVR can often be deferred into late childhood or adolescence. In our cohort, approximately two thirds of children remained free from AVR 10 years after their index repair.^12^ This delay is clinically meaningful because it allows the aortic root to reach a more stable, near-adult size before autograft implantation.

The principal limitation of repair is durability. At 15 years, freedom from aortic valve reoperation was significantly greater after the Ross procedure than after repair (70.7% vs 42.6%; *P* = .002).^11^ Advocates of an early Ross strategy emphasize that the pulmonary autograft functions as a living valve substitute, capable of somatic growth, which may reduce the cumulative burden of reintervention. More recent multicenter experience has also challenged historical concerns regarding neoaortic dilatation in young children, with long-term follow-up showing normalization of z scores in many patients.^13^ Although our series demonstrates superior survival after repair, several reports of the Ross procedure show survival comparable with that of the age-matched general population.^14^ Microsimulation analyses further suggest that the Ross procedure provides a survival advantage over mechanical or bioprosthetic AVR across the lifespan.^15^

The optimal timing of the Ross procedure therefore remains a topic of debate.^16^^,^^17^ A key observation from our institution supporting a repair-first approach is the favorable performance of the “secondary Ross,” performed after a previous native valve repair. Children undergoing a secondary Ross demonstrated superior 10-year survival (96.8% vs 90.0%; *P* = .04) and greater freedom from autograft reoperation (97.0% vs 82.0%; *P* = .03) compared with those undergoing a primary Ross.^18^ These findings suggest that initial native valve repair does not compromise subsequent autograft performance and may instead optimize conditions for a later Ross procedure by permitting continued somatic growth and stabilization of the aortic root before implantation. A related unresolved question is how best to manage failed native valve repair: whether re-repair should be attempted or whether the Ross procedure should be performed at that stage, and whether repeated repair attempts influence long-term outcomes. This remains an area of active investigation within our program.

## Truncal Valve Repair Versus Replacement

Management of significant truncal valve insufficiency in the neonate remains one of the greatest technical challenges in congenital cardiac surgery. Truncal valve morphology is highly variable and often dysplastic; quadricuspid anatomy is present in approximately 30% to 40% of cases and is the configuration most commonly associated with regurgitation. Although overall outcomes for truncus arteriosus repair have improved, moderate-to-severe truncal valve insufficiency continues to be a powerful independent risk factor for mortality.^19^ As pulmonary vascular resistance falls after birth, systemic output is diverted to the pulmonary circulation, and regurgitation further lowers diastolic pressure and impairs coronary perfusion. Establishing truncal valve competence is therefore essential to preserve myocardial viability.

The strategy for achieving competence remains debated. Neonatal truncal valve replacement has historically been associated with prohibitive early mortality, approaching 50% in many series, and prosthetic or homograft substitutes lack the capacity for growth.^20^ These limitations have prompted exploration of alternatives such as partial heart transplantation.^21^ However, whenever possible, we believe the primary objective should remain restoration of the child's native valve.

We regard most truncal valves as repairable, with the immediate goal of achieving a competent valve that permits safe stabilization during the vulnerable neonatal period and early infancy.^22^ Even when repair durability is limited, a later re-repair or replacement in childhood is generally safer and technically more predictable, and it allows implantation of larger and more durable material.^23^

We favor standardized, anatomy-guided reconstruction with attention to restoring root geometry and achieving reliable cusp coaptation.^4^^,^^22^ Quadricuspid valves, which are frequently regurgitant, are typically converted to a tricuspid configuration, and native cusp tissue is preserved whenever feasible. Active assessment of the coronary ostia forms an essential component of this strategy.^24^

In our experience, this reconstructive approach can restore adequate function to even severely dysplastic truncal valves in the neonatal period. Although long-term durability is ultimately determined by the quality of the cusp tissue, avoidance of neonatal valve replacement confers a substantial early survival advantage and remains, in our view, the preferred therapeutic objective.

## Discussion

Management of congenital aortic and truncal valve disease remains constrained by the absence of Level A evidence. The time horizon required to assess durability means that contemporary operative strategies will continue to evolve faster than long-term outcome data can accumulate. In this setting, clinical decision-making must rely on the best available evidence, including propensity-matched analyses, systematic reviews, and well-characterized longitudinal institutional series. Within this framework, our institutional experience has led to a bias in favor of reconstructive strategies, a position grounded in both the available data and fundamental biological principles of native tissue preservation and growth.

Across the 3 areas of equipoise discussed, a consistent theme emerges. In neonatal aortic stenosis, anatomy-guided surgical repair appears to provide superior durability compared with balloon valvuloplasty and can delay the need for aortic valve replacement until later childhood. In children with native aortic valve disease, initial valve repair offers a survival advantage and often serves as an effective bridge, with the option of a later Ross procedure when somatic growth is more advanced. For the neonatal truncal valve, strategies focused on preservation and reconstruction of the native valve—rather than primary replacement—offer the best opportunity to avoid the high early mortality associated with neonatal prosthetic valve implantation.

Taken together, these considerations support a gradual shift away from non-specific or palliative interventions toward precise, anatomy-based reconstructions that preserve native tissue whenever feasible. Although the rarity and heterogeneity of these lesions will continue to limit the strength of available evidence, the accumulating experience with standardized repair strategies suggests that native valve preservation not only delays valve replacement but may improve outcomes across the patient's life course.

### Webcast

You can watch a Webcast of this AATS meeting presentation by going to: https://www.aats.org/resources/congenital-aortic-and-truncal--10219.

**Figure:**
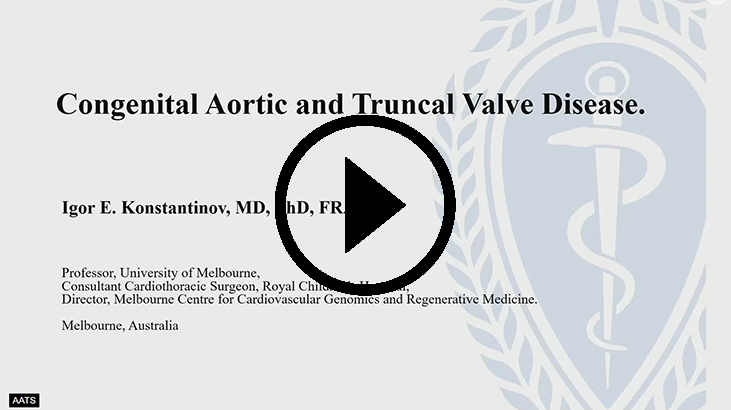

## Conflict of Interest Statement

The authors reported no conflicts of interest.

The *Journal* policy requires editors and reviewers to disclose conflicts of interest and to decline handling or reviewing manuscripts for which they may have a conflict of interest. The editors and reviewers of this article have no conflicts of interest.
